# Non-invasive peripheral vascular function, incident cardiovascular disease, and mortality in the general population

**DOI:** 10.1093/cvr/cvab087

**Published:** 2021-03-16

**Authors:** Renate B Schnabel, Christina Magnussen, Andreas Schulz, Francisco M Ojeda, Volker H Schmitt, Natalie Arnold, Christoph R Sinning, Manfred E Beutel, Irene Schmidtmann, Norbert Pfeiffer, Anja Leuschner, Karl J Lackner, Tommaso Gori, Emelia J Benjamin, Harald Binder, Philipp S Wild, Stefan Blankenberg, Thomas Münzel, for the Gutenberg Health Study investigators

**Affiliations:** 1 Department of Cardiology, University Heart and Vascular Center Hamburg, Martinistrasse 52, 20246 Hamburg, Germany; 2 German Center for Cardiovascular Research (DZHK), partner site Hamburg/Kiel/Luebeck, Germany; 3 Preventive Cardiology and Preventive Medicine, Center for Cardiology, University Medical Center Mainz, Langenbeckstraße 1, 55131 Mainz, Germany; 4 Department of Cardiology – Cardiology I, University Medical Center, Johannes Gutenberg University Mainz, Langenbeckstr. 1, 55131 Mainz, Germany; 5 German Center for Cardiovascular Research (DZHK), partner site Rhine/Main, Mainz, Germany; 6 Department of Psychosomatic Medicine and Psychotherapy, University Medical Center Mainz, Langenbeckstraße 1, 55131 Mainz, Germany; 7 Institute of Medical Biostatistics, Epidemiology and Informatics (IMBEI), University Medical Center Mainz, Langenbeckstraße 1, 55131 Mainz, Germany; 8 Department of Ophthalmology, University Medical Center Mainz, Langenbeckstraße 1, 55131 Mainz, Germany; 9 Institute of Clinical Chemistry and Laboratory Medicine, University Medical Center Mainz, Mainz, Germany; 10 NHLBI’s and Boston University’s Framingham Study, Framingham Heart Study 73 Mt. Wayte Avenue, Suite 2, Framingham, MA 01702-5827, USA; 11 Epidemiology Department, School of Public Health, Boston University, 715 Albany Street The Talbot Building, T3E & T4E, Boston, MA 02118, USA; 12 Whitaker Cardiovascular Institute, Evans Memorial Medicine Department, and Sections of Cardiology, and Preventive Medicine, Boston University School of Medicine, Boston, MA; 13 Center for Thrombosis and Hemostasis, University Medical Center Mainz, Mainz, Germany

**Keywords:** Mortality, Peripheral arterial tonometry, Non-invasive vascular function, Flow-mediated dilatation, Epidemiology, Cohort

## Abstract

**Aims:**

Evidence suggests that peripheral vascular function is related to cardiovascular disease (CVD) and mortality. We evaluated the associations of non-invasive measures of flow-mediated dilatation and peripheral arterial tonometry with incident CVD and mortality.

**Methods and results:**

In a *post-hoc* analysis of the community-based Gutenberg Health Study, median age 55 years (25th/75th percentile 46/65) and 49.5% women, we measured brachial artery flow-mediated dilatation (*N*=12 599) and fingertip peripheral arterial tonometry (*N*=11 125). After a follow-up of up to 11.7 years, we observed 595 incident CVD events, 106 cardiac deaths, and 860 deaths in total. Survival curves showed decreased event-free survival with higher mean brachial artery diameter and baseline pulse amplitude and better survival with higher mean flow-mediated dilatation and peripheral arterial tonometry ratio (all *P*_log rank _<0.05). In multivariable-adjusted Cox regression analyses only baseline pulse amplitude was inversely related to mortality [hazard ratio (HR) per standard deviation increase, 0.86, 95% confidence interval (95% CI), 0.79–0.94; *P*=0.0009]. After exclusion of individuals with prevalent CVD the association was no longer statistically significant in multivariable-adjusted models (HR 0.91, 95% CI 0.81–1.02; *P*=0.11). None of the vascular variables substantially increased the C-index of a model comprising clinical risk factors.

**Conclusions:**

In our cohort, non-invasive measures of peripheral vascular structure and function did not reveal clinically relevant associations with incident CVD or mortality. Whether determination of pulse amplitude by peripheral arterial tonometry improves clinical decision-making in primary prevention needs to be demonstrated.

## Graphical Abstract

**Figure:**
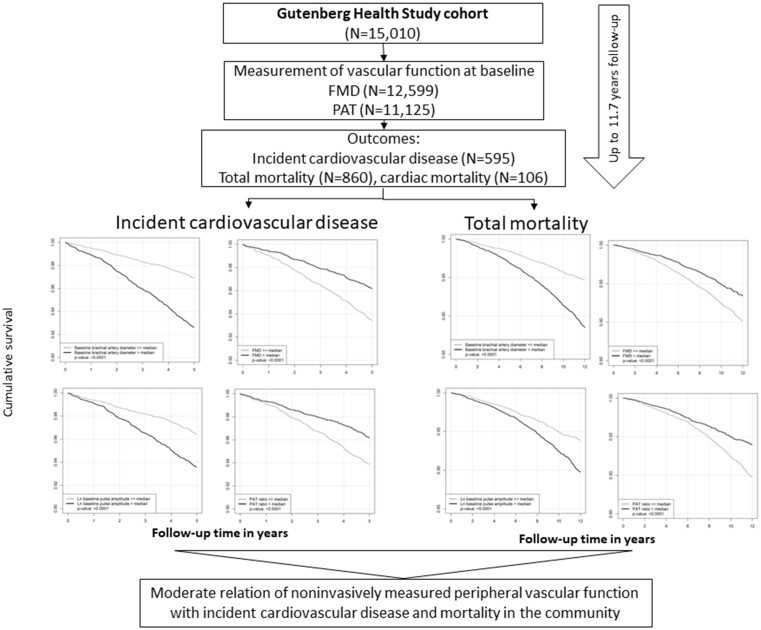

## 1. Introduction

Cardiovascular diseases (CVDs) are the major cause of mortality in aging populations worldwide. They are associated with vascular dysfunction that manifests at early, possibly reversible stages. The availability of nitric oxide plays a central role in vascular homeostasis and is determined by the functionality of the endothelium.^1^^,^^2^ Two non-invasive vascular function measurement methods have emerged in the past years, that reflect, at least in part, endothelial function and nitric oxide bioavailability: flow-mediated dilatation (FMD) and peripheral arterial tonometry (PAT).^3^^,^^4^ Both tests can be reliably applied at a large scale.^5–7^ Correlations with classical cardiovascular risk factors (CVRFs) have been reported in detail, but comparatively little is known on the prospective association of non-invasive vascular function testing with mortality. Whereas for FMD, an association with cardiovascular events and death in community cohorts has been shown,^8–10^ current evidence for the relation of PAT and mortality is limited.^11^ PAT has revealed discriminatory ability for the detection of attenuated coronary microvascular endothelial dysfunction,^12^ which is a predictor of cardiovascular events.^13^ A small longitudinal study in outpatients with unexplained chest pain revealed an association of endothelial dysfunction, detected by PAT with cardiovascular events and death.^14^ The relation of PAT to incident CVD and mortality in the general population is largely unknown.

To further elucidate the role of non-invasive vascular function measures and their potential clinical impact in the general population, we examined the association of PAT in comparison with FMD and classical CVRFs in relation to incident CVD overall and cardiovascular mortality in the community-based Gutenberg Health Study. We hypothesized that better vascular reactivity is inversely related to mortality.

## 2. Methods

### 2.1 Study sample

Individuals (*n*=15 010) were recruited in the Gutenberg Health Study cohort from 2007 to 2012 and were followed by a telephone interview and by an in-clinic visit after 5 years. All residents of the city of Mainz and the county Mainz/Bingen aged between 35 and 74 years were eligible. Participants were selected within age strata from a random sample through the local registration office. For the present investigation, we excluded individuals with missing vascular function measures, leaving 12 599 participants for FMD and 11 125 individuals for PAT analysis (84% and 74% of enrolled attendees) in our *post-hoc* analysis. The baseline characteristics of participants in whom vascular function measures were missing are provided in Supplementary material online, *Table S1*.

The project was approved by the local Ethics Committee. Participants provided written informed consent. The study adhered to all ethical principles for the good conduct of research with humans outlined by the Declaration of Helsinki. All authors have read and agreed to the manuscript as written. The data underlying this article will be shared on reasonable request to the corresponding author.

### 2.2 Risk factor assessment and follow-up

During the 5 h baseline clinic visit, comprehensive information on CVRFs was gathered by means of standardized interviews, anthropometric measures, and laboratory assessments. Assessment of current smoking, prevalent CVD, and medications relied on self-reported data. Smoking status included two categories: non-smokers (never smokers and former smokers) and current smokers. The diagnosis of diabetes comprised a physician’s diagnosis of diabetes and/or HbA1c≥6.5% and/or glucose lowering drugs (ATC code a10). Dyslipidaemia was defined as a physician’s diagnosis of dyslipidaemia and/or an LDL/HDL ratio of >3.5. Creatinine and blood lipids were measured immediately by routine methods. CVD comprised self-reported coronary artery disease, myocardial infarction, heart failure, or stroke.

Regular follow-ups were performed by the Gutenberg Health Study staff. For all-cause mortality official death certificates were acquired from the local registry offices. Participants’ health records information and death certificates were used to identify the occurrence of CVD events and the cause of death. Adjudication was performed by an expert team. CVD events comprises first acute myocardial infarct (ICD-10: I21), cerebral infarction/ischemic stroke (ICD-10: I63), atrial fibrillation (ICD I48), peripheral artery disease (ICD-10: I73.9), coronary artery disease (ICD-10: I25.10), heart failure (ICD-10: I50, I11.0, I13.0, I13.2), and sudden cardiac death (ICD-10: I46).

### 2.3 Vascular function measurement

Standardized non-invasive vascular function measurements were performed by experienced and certified technicians in dark, air-conditioned rooms after at least 5 min rest.^7^ Pneumatic pulse amplitude was measured using the Endo-PAT2000 fingertip device (Itamar Medical, Caesarea, Israel) under resting conditions and after induction of local reactive hyperaemia by upper arm occlusion. Pulse amplitude was recorded electronically in both index fingers. The left index finger served as the control finger. A computerized algorithm automatically generated the results. We excluded individuals if quality criteria were not met, including noisy signals (valid signals in the region of interest <80%), breakthrough of the arterial pulse curve during occlusion, and a >5.5 or <4.5 min occlusion duration.

High-resolution ultrasonic imaging of the right brachial artery by linear array broadband probe (L12–5; 38 mm) was performed on a Philips HD11XE CV ultrasound machine (Philips, Best, the Netherlands). Baseline loops and loops recorded 60 s after upper arm cuff release were saved digitally and analysed off-line using a commercially available software (Medical Imaging Applications LLC, Iowa City, Iowa). Reproducibility of measurements was as reported in the literature with a range of intra observer intra class correlation coefficients from 0.72 to 1.0 and inter observer intra class correlation coefficients from 0.62 to 0.95. Details on the vascular measurement methods used have been published earlier.^7^

### 2.4 Statistical analyses

Continuous data are presented as median and 25th/75th percentile and binary data as absolute and relative frequencies. The number of individuals who entered the analyses is indicated in the tables. Survival curves were generated via the Kaplan–Meier method for each vascular function measure ≤ and above the median. We used multivariable-adjusted Cox proportional hazards regression models to relate vascular function measures to time until fist CVD event, overall death or cardiac death. For analyses on incident CVD, individuals with prevalent CVD were excluded. Follow-up for CVD and cardiac death was censored at 5 years because information on these specific outcomes is only available for this time period. The proportional hazards assumption was examined using the methods of Grambsch and Therneau.^15^ No deviations from this assumption were found. For FMD measurements, baseline brachial artery diameter was accounted for. The models were adjusted for (i) age, sex, and (ii) additionally for current smoking, body mass index, systolic blood pressure, heart rate, hypertension treatment, diabetes, LDL/HDL cholesterol, and lipid treatment. Mortality models were additionally adjusted for prevalent CVD.

We calculated the C-statistic before and after adding vascular function measures to examine model discrimination with respect to the risk of incident CVD, total and cardiac death. We applied 1000 times bootstrap re-sampling to derive a 95% confidence interval (95% CI).^16^ We tested for potential interactions by age and sex and the prevalence of CVD.

Since the improved risk prediction is clinically most relevant in intermediate risk groups, we further examined associations in individuals based on SCORE Deutschland with a focus on participants at 10 year risk of fatal CVD 5–20%.^17^ We also provide information in the low risk group. For these analyses only participants aged 40 years or older were used. Individuals older than 65 years were treated as if they were 65 years old in the score computation.

### 2.5 Secondary analyses

Since prevalent CVD is a powerful determinant of mortality, we conducted secondary analyses excluding prevalent CVD (*N*=1946) from the population at risk.

In exploratory analyses, we plotted hazard ratios (HRs) for ln baseline pulse amplitude against age, obtained from a Cox model containing an interaction term between these two, to understand age-related variation in outcome associations.

Analyses were performed using R software, R version 3.5.1 (R Core Team, 2018. R: A language and environment for statistical computing. R Foundation for Statistical Computing, Vienna, Austria). We assumed a two-sided *P*<0.05 as statistically significant.

## 3. Results

### 3.1 Study sample

The baseline characteristics of the study sample and vascular function variables are shown in *Table 1*. The cohort consisted of middle-aged to older adults, median age 55 years, 49.5% women. During 9.0 years median follow-up (maximum 11.7 years), *N*=595 participants experienced an incident CVD event, *N*=992 participants died, and *N*=106 cardiac deaths occurred.

**Table 1:** Baseline characteristics of the study sample

	All *N*=15 010	Completeness (%)
Age (years)	55 (46, 65)	100
Women, No. (%)	7426 (49.5)	100
Body mass index (kg/m²)	26.6 (23.9, 30)	99.9
Systolic blood pressure (mm Hg)	130 (120, 142)	99.9
Heart rate (bpm)	68 (61.5, 75.5)	99.4
Current smoking, No. (%)	2911 (19.4)	99.8
Diabetes, No. (%)	1395 (9.3)	99.6
Dyslipidaemia, No. (%)	5176 (34.6)	99.7
Lipid-lowering medication, No. (%)	1984 (13.4)	99.0
Hypertension, No. (%)	7466 (49.8)	99.9
Antihypertensive medication, No. (%)	4430 (29.5)	99.9
Cardiovascular disease, No. (%)	1946 (13.1)	99.0
SCORE^a^	1.0 (0, 4.0)	99.7
Vascular function measures		
Baseline brachial artery diameter (mm)	4.3 (3.7, 4.9)	91.1
FMD (%)	7.3 (4.5, 10.9)	83.9
Ln baseline pulse amplitude (arbitrary units)	6.1 (5.4, 6.7)	74.1
PAT ratio	0.7 (0.3, 1)	74.1

Data are presented as median and 25th/75th percentile for continuous variables and *n* (percentage) for dichotomous variables. Completeness indicates the proportion of variables that were available for analysis.

FMD, flow-mediated dilatation; PAT, peripheral arterial tonometry.

a SCORE Deutschland was calculated in individuals without prevalent cardiovascular disease.

**Figure 1:**
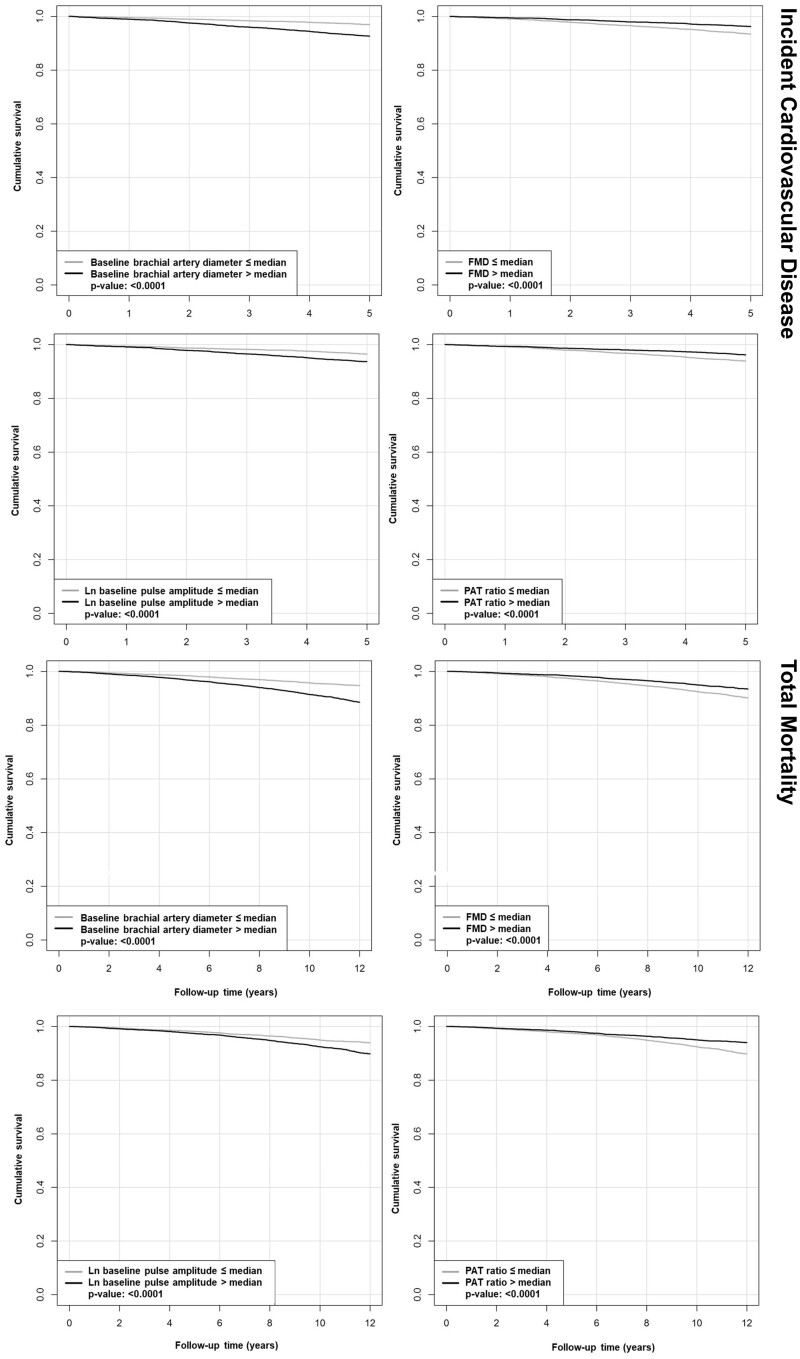
Survival curves stratified by median vascular function measures for baseline brachial artery diameter, FMD, ln baseline pulse amplitude, and PAT ratio for incident CVD (*N*=595) and total mortality (*N*=860). *P*-values are for the log rank test. FMD, flow-mediated dilatation; PAT, peripheral arterial tonometry.

Survival curves by median values of the vascular function measures showed higher event rates for incident CVD and death for higher baseline brachial artery and baseline pulse amplitude and lower risk with increased hyperaemic response (*Figure 1* and Supplementary material online, *Figure S1*).

**Figure 2:**
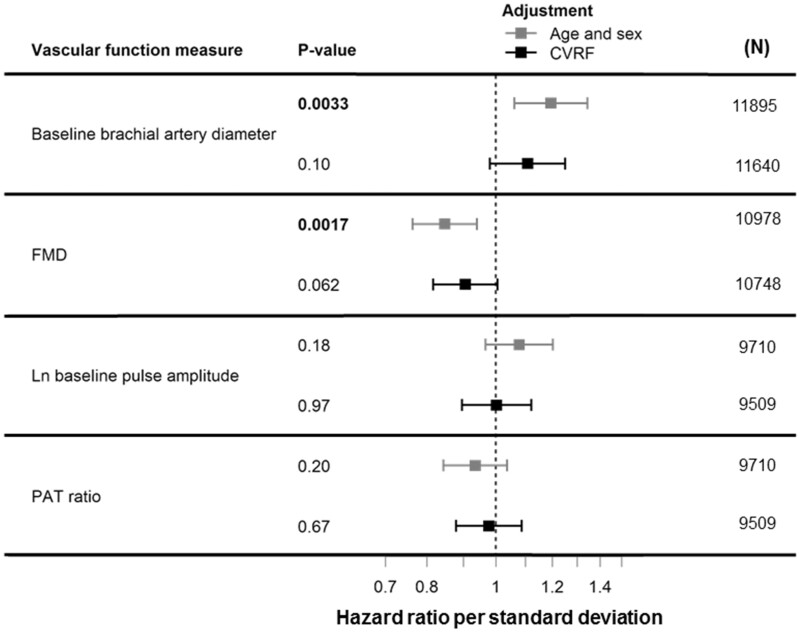
Vascular function measures in relation to incident CVD in multivariable-adjusted Cox regression analyses. Provided are HRs per SD increase in vascular function measure and 95% CI. Age- and sex-adjusted and CVRFs-adjusted models are presented. The latter include age, sex, current smoking, body mass index, systolic blood pressure, heart rate, hypertension treatment, diabetes, LDL/HDL cholesterol, lipid treatment, and prevalent CVD. FMD stands for flow-mediated dilatation, PAT for peripheral arterial tonometry.

*Figure 2* shows vascular function measures in relation to incident CVD in multivariable-adjusted Cox regression analyses. In age- and sex-adjusted analyses, baseline brachial artery diameter and FMD were statistically significantly associated with incident CVD (baseline brachial artery diameter HR 1.20; 95% CI 1.06–1.34; *P*=0.0033; HR for FMD 0.85; 95% CI 0.76–0.94; *P*=0.0017). Statistical significance was lost after further risk factor adjustment. The C-index did not improve compared to the clinical model (C-index 0.77; 95% CI 0.75–0.78), C-index clinical model and baseline brachial artery diameter 0.77; 95% CI 0.76–0.79, C-index clinical model and FMD 0.77; 95% CI 0.76–0.79.

*Table 2* provides HRs per standard deviation (SD) of vascular function variables for multivariable-adjusted associations with (i) total and (ii) cardiac mortality. In the multivariable-adjusted model, one SD increase in baseline pulse amplitude was associated with 14% lower mortality hazard (HR 0.86; 95% CI 0.79–0.94; *P*=0.0009). There was no significant association with cardiac mortality. The C-index of the basic model comprising all clinical risk factors used in the current analyses was 0.80 (95% CI, 0.788–0.813). The clinical model for mortality was not substantially improved by the addition of vascular function variables: brachial artery diameter C-index 0.802 (95% CI 0.790–0.817), FMD 0.804 (95% CI 0.790–0.819), baseline pulse amplitude 0.808 (95% CI 0.793–0.823), and PAT ratio 0.807 (95% CI 0.793–0.823).

**Table 2:** Vascular function measures in relation to (A) overall and (B) cardiac mortality in multivariable-adjusted Cox regression analyses

Variable	Sample	*N* events	HR (95% CI)	*P*-value
(A)				
Baseline brachial artery diameter, mm	13 671	893	1.01 (0.92, 1.10)	0.90
	13 370	865	0.96 (0.87,1.05)	0.33
FMD (%)	12 599	822	0.94 (0.89, 1.04)	0.29
	12 331	801	0.99 (0.91, 1.08)	0.87
Ln baseline pulse amplitude^a^	11 125	730	0.91 (0.84 0.99)	**0.026**
	10 890	712	0.86 (0.79,0.94)	**0.0009**
PAT ratio	11 125	730	0.95 (0.88,1.03)	0.22
	10 890	712	1.03 (0.94,1.12)	0.47
(B)				
Baseline brachial artery diameter, mm	13 245	106	1.21 (0.92, 1.60)	0.18
	12 958	102	1.05 (0.80, 1.38)	0.7
FMD (%)	12 204	98	0.81 (0.61, 1.07)	0.13
	11 950	95	0.95 (0.73, 1.24)	0.69
Ln baseline pulse amplitude^a^	10 784	84	0.924 (0.71, 1.19)	0.54
	10 561	81	0.84 (0.64, 1.10)	0.20
PAT ratio	10 784	84	0.85 (0.67, 1.07)	0.16
	10 561	81	1.03 (0.80, 1.33)	0.83

Provided are hazard ratios (HR) and 95% confidence intervals (95% CI) per standard deviation increase in vascular function measure. *P*-values <0.05 are printed in bold. FMD, flow-mediated dilatation; PAT, peripheral arterial tonometry.

Upper rows are age- and sex-adjusted models, lower rows are multivariable-adjusted models including age, sex, current smoking, body mass index, systolic blood pressure, heart rate, hypertension treatment, diabetes, LDL/HDL cholesterol, lipid treatment, and prevalent cardiovascular disease.

* Arbitrary units.

The baseline characteristics by SCORE risk category are available in Supplementary material online, *Table S2*, association analyses in Supplementary material online, *Figures S2–S7*. In the subgroup of individuals with intermediate risk of fatal CVD (*N*=3239, 21.6% women, median age 67 years), we observed *N*=268 incident CVD events, *N*=518 deaths. No statistically significant association was observed for incident CVD (Supplementary material online, *Figure S5*). Baseline pulse amplitude was the only vascular function measure statistically significantly related to the mortality in both, age- and sex as well as multiple risk factor-adjusted analyses (HR 0.80; 95% CI 0.70–0.91; *P*<0.001) (Supplementary material online, *Figure S6*). The C-indices were not improved by the addition of vascular function measurements to the clinical risk factors in the intermediate risk group (Supplementary material online, *Table S3*).

To further explore the unexpected negative correlation of baseline pulse amplitude and mortality, we calculated HRs adjusting for each CVRF separately (Supplementary material online, *Table S4*). We observed that the association of ln baseline pulse amplitude and mortality was positive in the unadjusted model and remained positive when adjusting for each CVRF separately. When both age and sex were entered into the model the sign of the beta changed. Formal interaction testing for sex and age did not reach statistical significance. However, an interaction of the association by prevalent CVD was statistically significant, *P*=0.0032. We further examined the association of baseline pulse amplitude with mortality by age. In Supplementary material online, *Figure S8*, we plotted HRs against increasing age. Below the age of about 65 years, we observed a positive association with mortality whereas above this age, the association changed. The 95% CI crossed 1.0 for all associations.

### 3.2 Secondary analyses

In secondary analyses, the exclusion of individuals with prevalent CVD at baseline (for baseline characteristics see Supplementary material online, *Table S5*) resulted in comparable associations in relation to mortality (*N*=563 deaths) in multivariable-adjusted models, but ln baseline pulse amplitude did not show statistical significance (HR 0.90, 95% CI 0.81–1.01; *P*=0.068) (Supplementary material online, *Table S6*).

## 4. Discussion

### 4.1 Principal findings

In our large contemporary community-based cohort, we observed a moderate relation of non-invasively measured vascular function with incident CVD and mortality, which was largely explained by classical CVRFs. Whereas an inverse association of baseline pulse amplitude was seen in the overall cohort, no statistically significant association with mortality could be demonstrated after exclusion of individuals with prevalent CVD. Risk prediction was not substantially improved.

Two non-invasive methods of vascular function testing have gained popularity over the past years.^18^ We have viewed FMD and PAT as possible markers of cardiovascular morbidity and indicators of vascular ageing. The only vascular variable related to outcome in multivariable-adjusted analyses was baseline pulse amplitude. Whereas in unadjusted models, the cumulative incidence of deaths increased with higher baseline pulse amplitude, it showed a counterintuitive risk reduction in multivariable models. In univariate and risk factor-adjusted models resulted in a positive correlation with mortality as expected from pathophysiology. When both, age and sex were entered into the model, the sign switched indicating a possible interaction. In younger age groups an increased risk of death was observed in relation to higher pulse amplitude, whereas in older individuals above the age of 65 years the HRs appeared to be protective. In the intermediate risk group with a SCORE Deutschland between 5% and 20%, 78.4% of participants were men with a median age of 67 years, which may help explain the association observed in this subgroup. No statistically significant association of baseline pulse amplitude with cardiac and mortality was seen in participants without CVD. This fact may help explain our observations in the overall cohort where the interaction by prevalent CVD was statistically significant. Individuals with prevalent CVD usually are older, more frequently men and have a higher risk factor burden resulting in a higher mortality. When entering CVD into the equation, the association with baseline pulse amplitude switched to a positive association, as expected. In addition, in the Framingham Heart Study, a small positive association of PAT ratio with older age has been observed.^6^ An inverted or at least attenuated association of higher blood pressure and pulse amplitude and mortality in older individuals is known.^19^^,^^20^ Whether such observations help to explain our findings from a pathophysiological perspective is not clear.

The reasons for the weak and null findings are uncertain. A general endothelial function and nitric oxide dependence have consistently been demonstrated for PAT measures.^21^^,^^22^ However, it needs to be critically considered, that PAT determined at the fingertip relies on the measurement in a fine-tuned and rapidly changing vascular environment.^23^ PAT results from a complex interplay of digital microvessel dilatation, systemic and peripheral resistance, digital flow embedded in current autonomic state of activity, environmental conditions, and skin temperature. Quick alterations in vascular tone and reactivity will automatically lead to variability in measurement results despite high standardization of environmental conditions and measurement technique in our cohort. Thus, multiple factors acutely determine PAT, which may obscure long-term associations.

The assessment of the hyperaemic response after 5 min upper arm occlusion as an endothelial stress test did not enhance the predictive power over clinical models. Upper arm cuff occlusion tends to cause larger dilatations due to a range of different mechanisms, in particular short-term adaptions to ischaemia. The hyperaemic response therefore mirrors vascular changes beyond shear stress compared to forearm cuff occlusion and results are not directly comparable to prior FMD outcome studies. The exact implications of pulse amplitude measurement for clinical decision-making need to be evaluated. A correlation of baseline pulse amplitude with classical CVRFs, some of which are modifiable, has been established.^6^ Thus, it is likely that a modification of risk factors may improve peripheral pulse amplitude. The latter needs to be demonstrated.

Furthermore, we and others, have described differences in vascular function by sex with overall more beneficial vascular characteristics in women.^24^^,^^25^ However, we did not observe differential associations with mortality in sex-stratified subgroups or by interaction analyses for the examined vascular function measures. Baseline pulse amplitude was related to total mortality in men and women whereas none of the other vascular variables showed significant associations with outcome.

The correlation of PAT and FMD vascular function measures has been demonstrated to be weak.^7^^,^^24^ They represent different vascular beds, but neither baseline brachial artery diameter nor FMD showed an association with death. In prior work, it has been demonstrated that baseline brachial artery diameter is characterized by a weak to moderate correlation with classical CVRFs, whereas FMD is only weakly related to known risk factors.^5^^,^^7^^,^^26^^,^^27^ The proportion of variability explained by CVRFs is small. When we assume the endothelium to be a mirror of CVRF burden, no strong association with long-term CVD incidence and mortality can be expected. This assumption is in line with the current view of non-invasive vascular function measures, such as FMD and PAT as biological measures of vascular function rather than tests for clinical outcome prediction.

Our results for FMD appear to be consistent with a recent publication that examined brachial FMD in relation to incident CVD and mortality in the community-based Multi-Ethnic Study of Atherosclerosis in intermediate risk individuals.^9^ The association with the combined endpoint was only significant in univariable analysis and lost statistical significance after risk factor adjustment. No improvement in the area under the curve beyond traditional risk factors combined in the Framingham risk score was observed.

The study by Yeboah *et al.* was also included in a meta-analysis that analysed thirty-six studies using FMD for outcome prediction with an inclusion of 15 191 individuals.^28^ The authors found an overall relative risk of 0.90 per 1% increase in FMD. The majority of studies incorporated in the meta-analysis were small and comprised selected individuals with high risk factor burden or manifest disease. Thus, the comparability with data acquired in a uniform fashion in our middle-aged to older cohort is limited. They indicate that FMD measurements in specific groups of pre-defined individuals and secondary prevention may be higher compared to the general community.

### 4.2 Strengths and limitations

Strengths of the study are the standardized availability of non-invasive vascular function measures in one of the largest population-based samples. Standard operating procedures for FMD and PAT acquisition followed technical considerations outlined by Flammer *et al.*^18^ We have near complete follow-up. Routine ascertainment of potential clinical confounders provides the opportunity to carefully adjust confounders. As a limitation, we were not able to assess the flow stimulus or endothelial-independent vasoreactivity, which may deliver important additional information on vasofunction. Furthermore, the number of outcomes was moderate, which may have obscured weak associations.

Our data provide sound evidence on a moderate age- and sex-adjusted association of peripheral pulse amplitude with total mortality and no significant association with incident CVD or cardiac or total mortality for FMD after risk factor adjustment, applying two techniques of non-invasive vascular function testing in the Gutenberg Health Study. The clinical implication of our results is that unselected, routine measurement of PAT or FMD to assess CVD or mortality risk in the community appears not to be appropriate. The value of non-invasive peripheral function determination in selected subgroups or in the experimental setting needs to be examined.

## Supplementary material

Supplementary material is available at *Cardiovascular Research* online.

## Authors’ contributions

R.B.S.: drafting of the work, acquisition of funding, and interpretation of data for the work. C.M.: interpretation of data for the work and critical revision of the manuscript for important intellectual content. A.S. and F.M.O: analysis, or interpretation of data for the work. V.H.S. and N.A.: critical revision of the manuscript for important intellectual content. C.R.S.: substantial contributions to the acquisition of the data. M.E.B. and K.J.L.: substantial contributions to the conception or design of the work and critical revision. I.S., N.P., A.L., T.G., and H.B.: critical revision of the manuscript for important intellectual content. E.J.B.: substantial contributions to the conception or design of the work and data interpretation. P.S.W., S.B., and T.M.: substantial contributions to the conception or design of the work and acquisition of funding. All authors are accountable for parts/all aspects of the work.

## Supplementary Material

cvab087_Supplementary_Data

## Notes

Translational perspective In our large middle-aged community cohort with more than 15 000 individuals, median age 55 years (25th/75th percentile 46/65), 49.5% women non-invasively measured peripheral vascular function using flow-mediated dilatation after upper arm occlusion or fingertip peripheral arterial tonometry was not relevantly associated with incident cardiovascular disease or mortality in multivariable-adjusted analyses. An interaction of the association of peripheral arterial tonometry with mortality by prevalent cardiovascular disease was observed. Routine measurement of flow-mediated dilatation or peripheral arterial tonometry in our community cohort to screen for high risk of cardiovascular disease or mortality was not effective.
