# Supplementary material for: Non-invasive peripheral vascular function, incident cardiovascular disease, and mortality in the general population
Source: Cardiovasc Res. 2021 Mar 16;118(3):904–12. doi: 10.1093/cvr/cvab087 (PMC8859627; doi:10.1093/cvr/cvab087)

Noninvasive Peripheral Vascular Function, Incident Cardiovascular Disease and Mortality in the General Population

Renate B. Schnabel, MD, MSc; Christina Magnussen, MD; Andreas Schulz; Francisco M. Ojeda, PhD; Volker H. Schmitt, MD; Natalie Arnold, MD; Christoph R. Sinning, MD; Manfred E. Beutel, MD; Irene Schmidtmann, PhD; Norbert Pfeiffer, MD; Anja Leuschner, MD; Karl J. Lackner, MD; Tommaso Gori, MD; Emelia J. Benjamin, MD, ScM; Harald Binder, PhD; Philipp S. Wild, MD; Stefan Blankenberg, MD; Thomas Münzel, MD, FAHA for the Gutenberg Health Study investigators

**Supplementary Table 1**	Baseline characteristics of individuals with vascular function measurements available and individuals with missing vascular function information	Page 2
**Supplementary Table 2**	Baseline characteristics of individuals with intermediate risk of cardiovascular death	Page 3
**Supplementary Table 3**	C-indices in intermediate risk individuals	Page 4
**Supplementary Table 4**	Selected Cox models with ln baseline pulse amplitude	Page 5
**Supplementary Table 5**	Baseline characteristics by prevalent cardiovascular disease status	Page 6
**Supplementary Table 6**	Cox regression analyses in individuals without cardiovascular disease	Page 7
**Supplementary Figure 1**	Survival curves stratified by median vascular function measures	Page 8
**Supplementary Figure 2**	Vascular function measures in relation to outcomes by risk category	Page 9
**Supplementary Figure 3**	Hazard ratios of baseline pulse amplitude plotted against age.	Page 15

Supplementary Table 1.

Variable	FMD N=12599	FMD missing N=2411	PAT N=11125	PAT missing N=3885
Age, years	55 (45/64)	57 (48/66)	55 (45/64)	57 (47/66)
Women, % (No)	48.9 (6156/12599)	52.7 (1270/2411)	48.5 (5394/11125)	52.3 (2032/3885)
BMI, (kg/m²)	26.6 (23.9/29.9)	26.9 (24.1/31.1)	26.5 (23.9/29.8)	26.8 (24.0/30.9)
Current smoking, % (No)	19.6 (2463/12576)	18.7 (448/2399)	19.5 (2162/11105)	19.4 (749/3870)
Diabetes, % (No)	9.1 (1142/12563)	10.6 (253/2392)	9.0 (998/11093)	10.3 (397/3862)
Dyslipidemia, % (No)	34.4 (4328/12570)	35.4 (848/2395)	34.2 (3802/11103)	35.6 (1374/3862)
Lipid-lowering medication,% (No)	13.1 (1629/12466)	14.9 (355/2390)	12.9 (1418/11001)	14.7 (566/3855)
Hypertension, % (No)	48.9 (6160/12594)	54.3 (1306/2407)	48.8 (5433/11122)	52.4 (2033/3879)
Antihypertensive medication, % (No)	28.6 (3596/12592)	34.7 (834/2401)	28.5 (3168/11120)	32.6 (1262/3873)
Cardiovascular disease, % (No)	12.7 (1584/12486)	15.2 (362/2376)	12.4 (1368/11027)	15.1 (578/3835)
Systolic blood pressure (mm Hg)	129.5 (119/141.5)	131 (121/144)	129.5 (119/141.5)	130.5 (120/143)
Heart rate (bpm)	68 (61.5/75.5)	68.5 (62/75.7)	68 (61.5/75.5)	68 (61.5/76)

Data are presented as median and 25th/75th percentile for continuous variables and percentage (numbers with condition/total number) for dichotomous variables.

BMI stands for body mass index.

Supplementary Table 2. Baseline characteristics of individuals with intermediate 10-year risk of cardiovascular death

Variable	Low risk group N=11,680	Intermediate risk group N=3,239	High risk group N=20
Age (years)	51 (44, 59)	67 (63, 70)	67 (65/70)
Women No. (%)	6680 (57.2)	701 (21.6)	0
BMI (kg/m²)	26.3 (23.6, 29.9)	27.6 (25.2, 30.5)	28.5 (27.0, 30.5)
Current smoking No. (%)	2164 (18.5)	720 (22.2)	20 (100)
Diabetes No. (%)	828 (7.1)	548 (16.9)	7 (35)
Dyslipidemia No. (%)	3555 (30.5)	1588 (49.1)	10 (50)
Lipid-lowering medication No. (%)	1246 (10.8)	725 (22.5)	1 (5)
Hypertension No. (%)	4828 (41.3	2580 (79.7)	20 (100)
Antihypertensive medication No. (%)	2802 (24)	1593 (49.2)	8 (40)
Cardiovascular disease No. (%)	1247 (10.7)	684 (21.5)	1 (5)
Systolic blood pressure (mm Hg)	126 (117, 136.5)	145 (134, 157)	173 (155.2,177.3)
Heart rate (bpm)	68.5 (62.0, 75.5)	67.5 (60.5, 75.5)	77.0 (64.4, 82.5)
Vascular function measures			
Baseline brachial artery diameter (mm)	4.15 (3.6, 4.8)	4.9 (4.4, 5.4)	5.2 (4.55, 5.4)
FMD (%)	7.75 (4.7, 11.6)	6.1 (4, 8.7)	8.6 (5.5, 12.7)
Ln baseline pulse amplitude (arbitrary units)	6.0 (5.3, 6.6)	6.4 (5.8, 6.9)	6.7 (6.3, 7.2)
PAT ratio	0.7 (0.4/0.96)	0.5 (0.2, 0.8)	0.7 (0.2, 0.2)

Data are presented as median and 25th/75th percentile for continuous variables and n (percentage) for dichotomous variables.

Abbreviations: BMI, body mass index; FMD, flow mediated dilatation; PAT, peripheral arterial tonometry.

Supplementary Table 3. C-indices for the clinical model and vascular function measures in intermediate risk individuals based on SCORE Deutschland (N=3,239)

Model	**C-index (95% Confidence interval)**
Basic model	0.695 (0.661, 0.752)
Base model + baseline brachial artery diameter	0.700 (0.669, 0.758)
Base model + FMD	0.703 (0.672, 0.764)
Base model + ln baseline pulse amplitude	0.700 (0.671, 0.765)
Base model + PAT ratio	0.696 (0.664, 0.760)

Bootstrap analysis with 1000-fold re-sampling was used to correct for over-optimism.

The basic model comprised age, sex, current smoking, body mass index, systolic blood pressure, heart rate, hypertension treatment, diabetes, LDL/HDL cholesterol, lipid treatment, and prevalent cardiovascular disease.

Supplementary Table 4. Selected Cox models with ln baseline pulse amplitude

Beta (95% CI)	HR (95% confidence interval)	P Value	Predictors
0.4199 (0.3490, 0.4908)	1.52 (1.42, 1.63)	<0.001	ln baseline pulse amplitude
0.3967 (0.3012, 0.4921)	1.49 (1.35, 1.64)	<0.001	ln baseline pulse amplitude, male sex
0.3782 (0.3029, 0.4535)	1.46 (1.35, 1.57)	<0.001	ln baseline pulse amplitude, BMI, BMI^2
0.3113 (0.2376, 0.3850)	1.37 (1.27, 1.47)	<0.001	ln baseline pulse amplitude, Systolic BP, Antihypertensive medication
0.4369 (0.3658, 0.5081)	1.55 (1.44, 1.66)	<0.001	ln baseline pulse amplitude, heart rate
0.4246 (0.3532, 0.4961)	1.53 (1.42, 1.64)	<0.001	ln baseline pulse amplitude, current smoking
0.3879 (0.3146, 0.4611)	1.47 (1.37, 1.59)	<0.001	ln baseline pulse amplitude, LDL/HDL, $(LDL/HDL)^2$, lipid-lowering medication
0.3505(0.2781, 0.4228)	1.42 (1.32, 1.53)	<0.001	ln baseline pulse amplitude, diabetes
0.3472 (0.2746, 0.4198)	1.42 (1.32, 1.52)	<0.001	ln baseline pulse amplitude, cardiovascular disease
0.1962 (0.1228, 0.2696)	1.22 (1.13, 1.31)	<0.001	ln baseline pulse amplitude, age
-0.0014 (-0.0998, 0.0969)	1.00 (0.91, 1.10)	0.98	ln baseline pulse amplitude, age, male sex

Beta coefficients and hazard ratios (95% confidence interval) are provided. CI stands for confidence interval.

Supplementary Table 5. Baseline characteristics by prevalent cardiovascular disease status

Variable	No CVD N=12916	Prevalent CVD N=1946
Age (years)	54.0 (45.0/63.0)	63.0 (53.0/69.0)
Women, % (No.)	51.9 (6703/12916)	34.1 (663/1946)
BMI (kg/m²)	26.4 (23.7/29.7)	28.3 (25.2/31.5)
Current smoking, % (No.)	19.6 (2526/12900)	18.8 (365/1941)
Diabetes, % (No.)	7.6 (985/12876)	19.2 (373/1939)
Dyslipidemia, % (No.)	31.2 (4021/12888)	55.7 (1081/1941)
Lipid-lowering medication, % (No.)	9.5 (1214/12780)	37.9 (731/1928)
Hypertension, % (No.)	47.0 (6063/12909)	67.3 (1309/1945)
Antihypertensive medication, % (No.)	26.0 (3359/12914)	51.3 (998/1945)
Systolic blood pressure (mm Hg)	129 (119/141)	133 (122/145.5)
Heart rate (bpm)	68 (62/75.5)	67.5 (60/76)
CVD risk SCORE	1.0 (0/4.0)	3.0 (1.0/6.0)
Vascular function measures		
Baseline brachial artery diameter (mm)	4.27 (3.64/4.90)	4.69 (4.07/5.21)
FMD (%)	7.52 (4.67/11.13)	6.24 (3.90/9.34)
Ln baseline pulse amplitude (arbitrary units)	6.05 (5.33/6.69)	6.38 (5.71/6.87)
PAT ratio	0.68 (0.35/0.95)	0.49 (0.19/0.84)

Data are presented as median and 25th/75th percentile for continuous variables and percentage (numbers) for dichotomous variables.

Abbreviations: BMI, body mass index; CVD cardiovascular disease; FMD, flow mediated dilatation; PAT, peripheral arterial tonometry.

Supplementary Table 6. Vascular function measures in relation to mortality in multivariable-adjusted Cox regression analyses in individuals without manifest cardiovascular disease, N=12916

Variable	Hazard ratio per standard deviation (95% confidence interval)	P value	N events
Baseline brachial artery diameter, mm	0.95 (0.84, 1.07)	0.41	563
FMD, %	0.99 (0.89, 1.10)	0.82	519
Ln baseline pulse amplitude	0.90 (0.81, 1.01)	0.068	470
PAT ratio	1.0 (0.91, 1.11)	0.94	470

All models adjusted for age, sex, current smoking, body mass index, systolic blood pressure, heart rate, hypertension treatment, diabetes, LDL/HDL cholesterol and lipid treatment.

Supplementary Figure 1. Survival curves stratified by median vascular function measures for baseline brachial artery diameter, FMD, ln baseline pulse amplitude, and PAT ratio for cardiac mortality (N=106). P-values are for the log rank test.

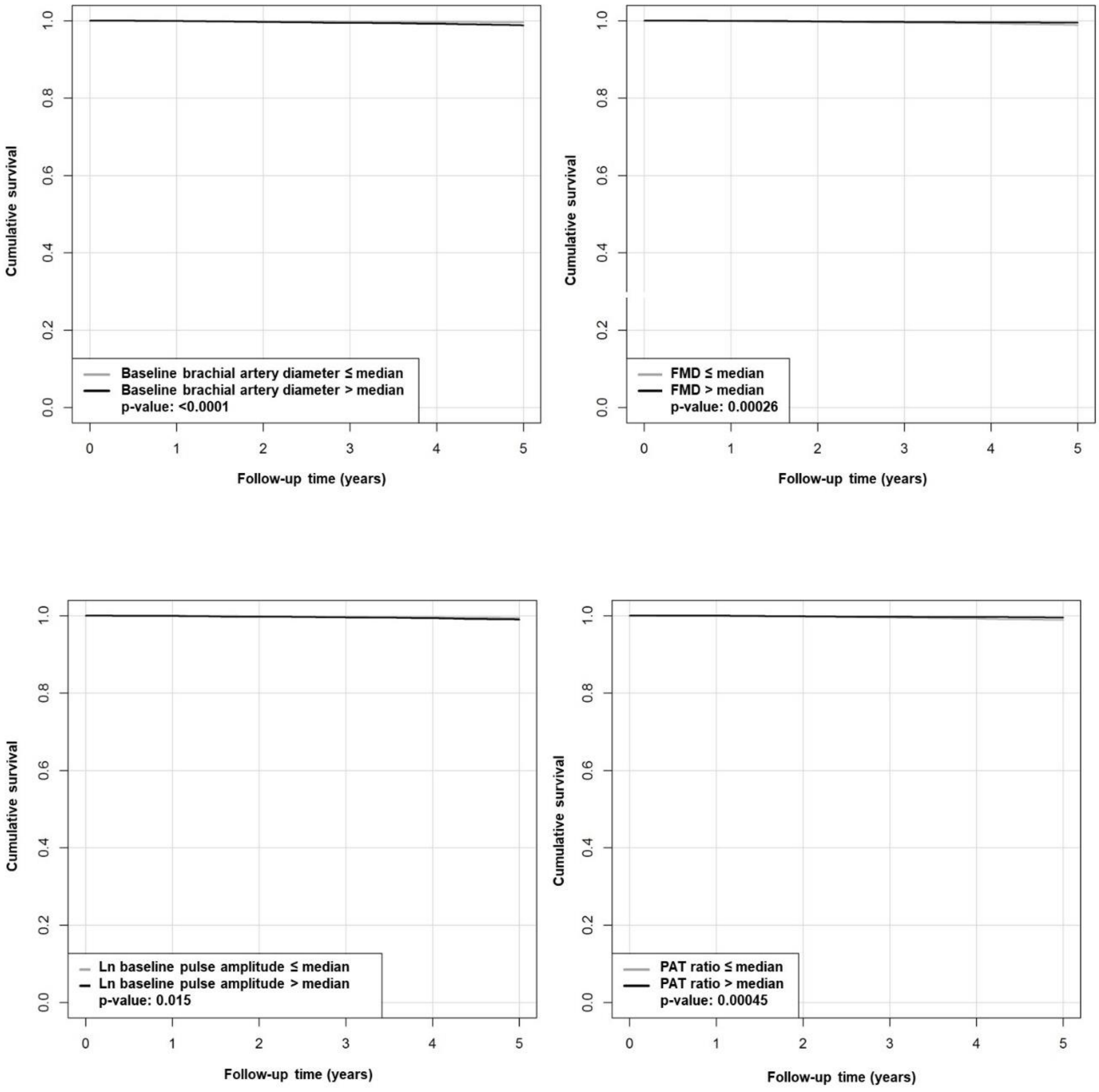

Supplementary Figure 2. Vascular function measures in relation to incident cardiovascular disease in multivariable-adjusted Cox regression analyses in the subgroup of individuals with a low ten-year risk of fatal cardiovascular disease (318 events). Provided are hazard ratios per standard deviation increase in vascular function measure and 95% confidence intervals. Hazard ratios (HR) are per standard deviation (SD).

Age- and sex-adjusted and cardiovascular risk factors (CVRF)-adjusted models are presented. The latter include age, sex, current smoking, body mass index, systolic blood pressure, heart rate, hypertension treatment, diabetes, LDL/HDL cholesterol, and lipid treatment. FMD stands for flow-mediated dilatation, PAT for peripheral arterial tonometry.

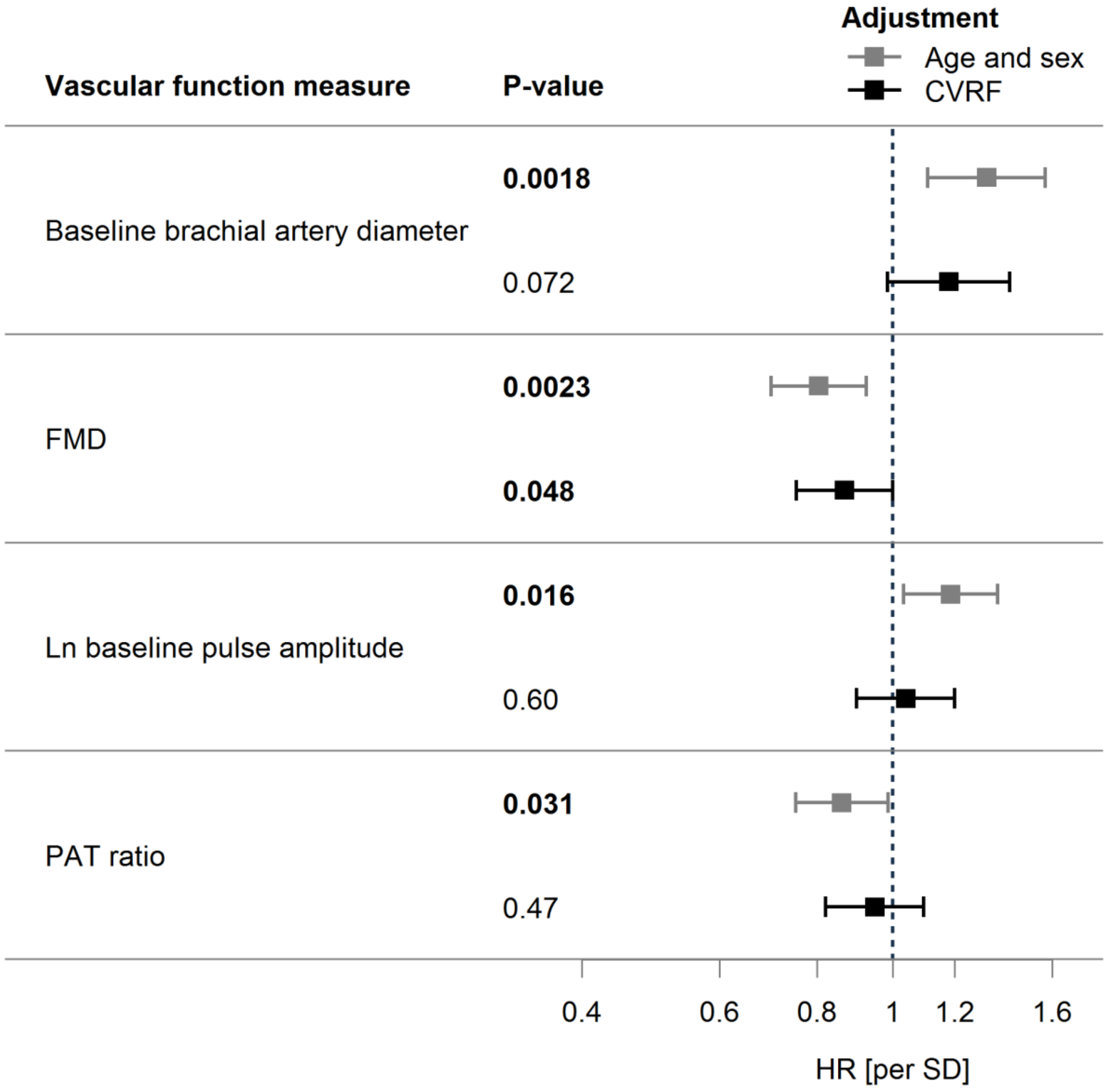

Supplementary Figure 3. Vascular function measures in relation to total mortality in multivariable-adjusted Cox regression analyses in the subgroup of individuals with a low ten-year risk of fatal cardiovascular disease (459 events). Provided are hazard ratios per standard deviation increase in vascular function measure and 95% confidence intervals. Hazard ratios (HR) are per standard deviation (SD).

Age- and sex-adjusted and cardiovascular risk factors (CVRF)-adjusted models are presented. The latter include age, sex, current smoking, body mass index, systolic blood pressure, heart rate, hypertension treatment, diabetes, LDL/HDL cholesterol, and lipid treatment. FMD stands for flow-mediated dilatation, PAT for peripheral arterial tonometry.

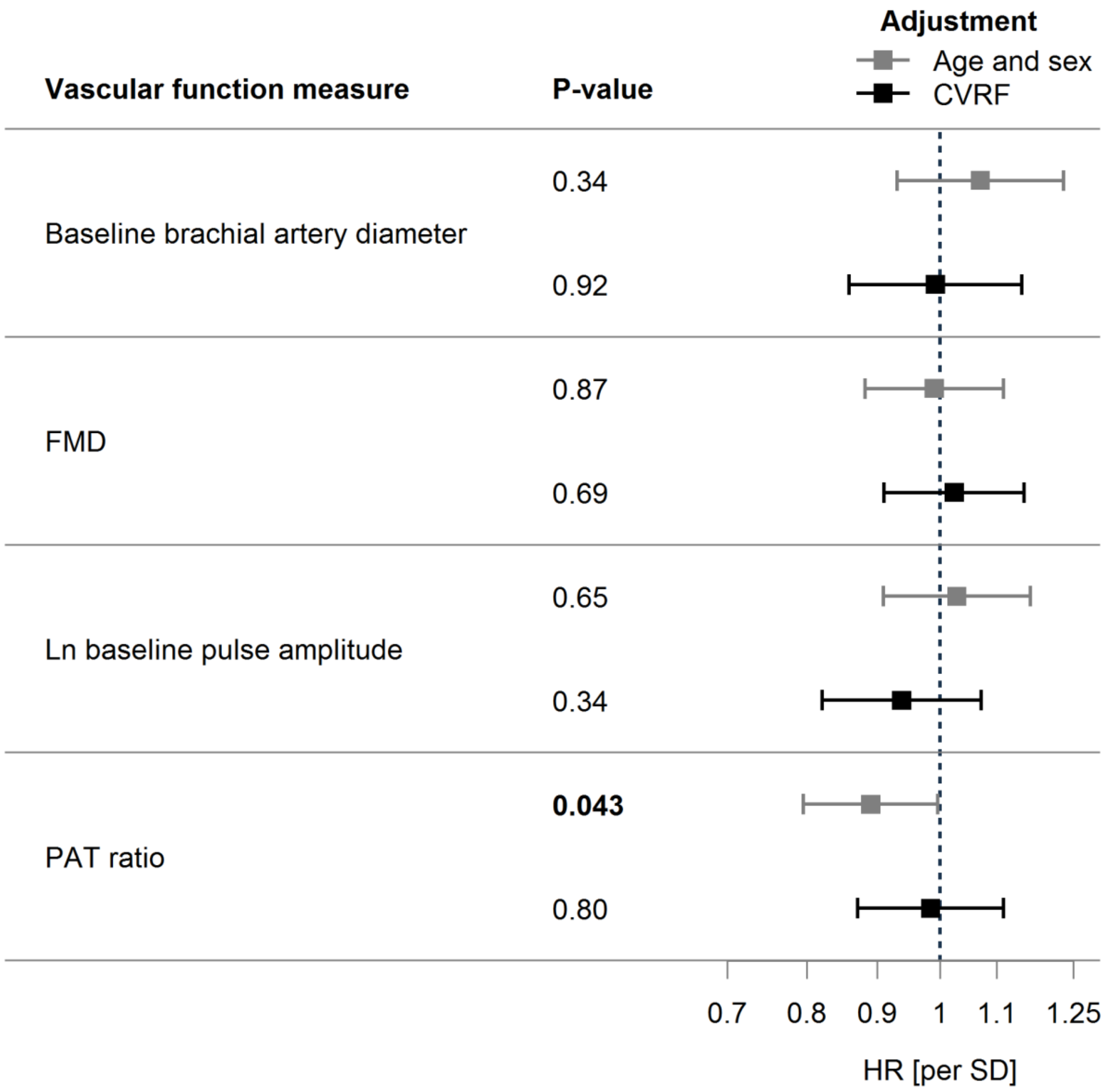

Supplementary Figure 4. Vascular function measures in relation to cardiac mortality in multivariable-adjusted Cox regression analyses in the subgroup of individuals with a low ten-year risk of fatal cardiovascular disease (45 deaths). Provided are hazard ratios (HR) per standard deviation (SD) increase in vascular function measure and 95% confidence intervals.

Age- and sex-adjusted and cardiovascular risk factors (CVRF)-adjusted models are presented. The latter include age, sex, current smoking, body mass index, systolic blood pressure, heart rate, hypertension treatment, diabetes, LDL/HDL cholesterol, lipid treatment, and prevalent cardiovascular disease. FMD stands for flow-mediated dilatation, PAT for peripheral arterial tonometry.

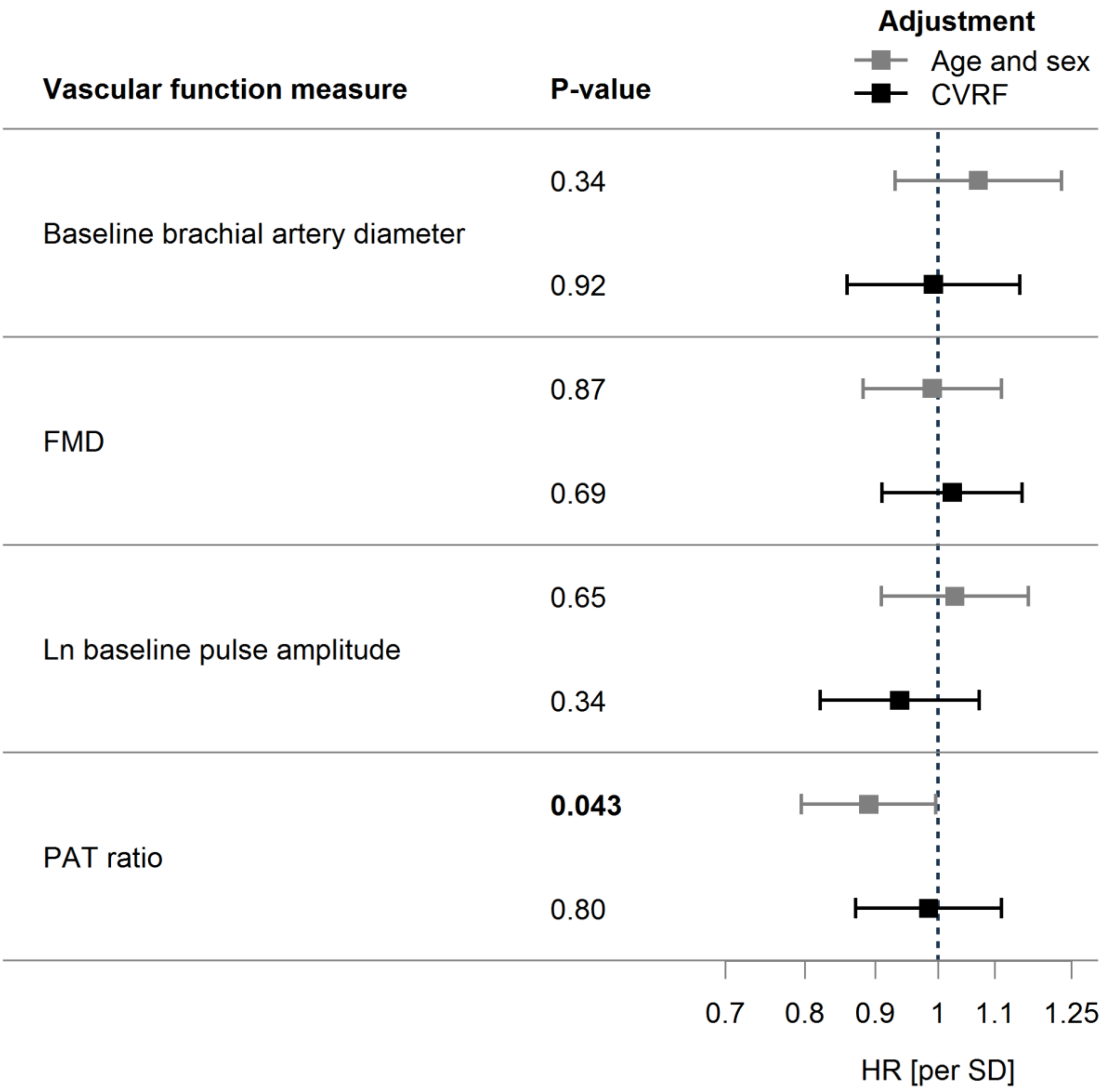

Supplementary Figure 5. Vascular function measures in relation to incident cardiovascular disease in multivariable-adjusted Cox regression analyses in the subgroup of individuals with an intermediate ten-year risk of fatal cardiovascular disease (268 events). Provided are hazard ratios (HR) per standard deviation (SD) increase in vascular function measure and 95% confidence intervals.

Age- and sex-adjusted and cardiovascular risk factors (CVRF)-adjusted models are presented. The latter include age, sex, current smoking, body mass index, systolic blood pressure, heart rate, hypertension treatment, diabetes, LDL/HDL cholesterol, lipid treatment, and prevalent cardiovascular disease. FMD stands for flow-mediated dilatation, PAT for peripheral arterial tonometry.

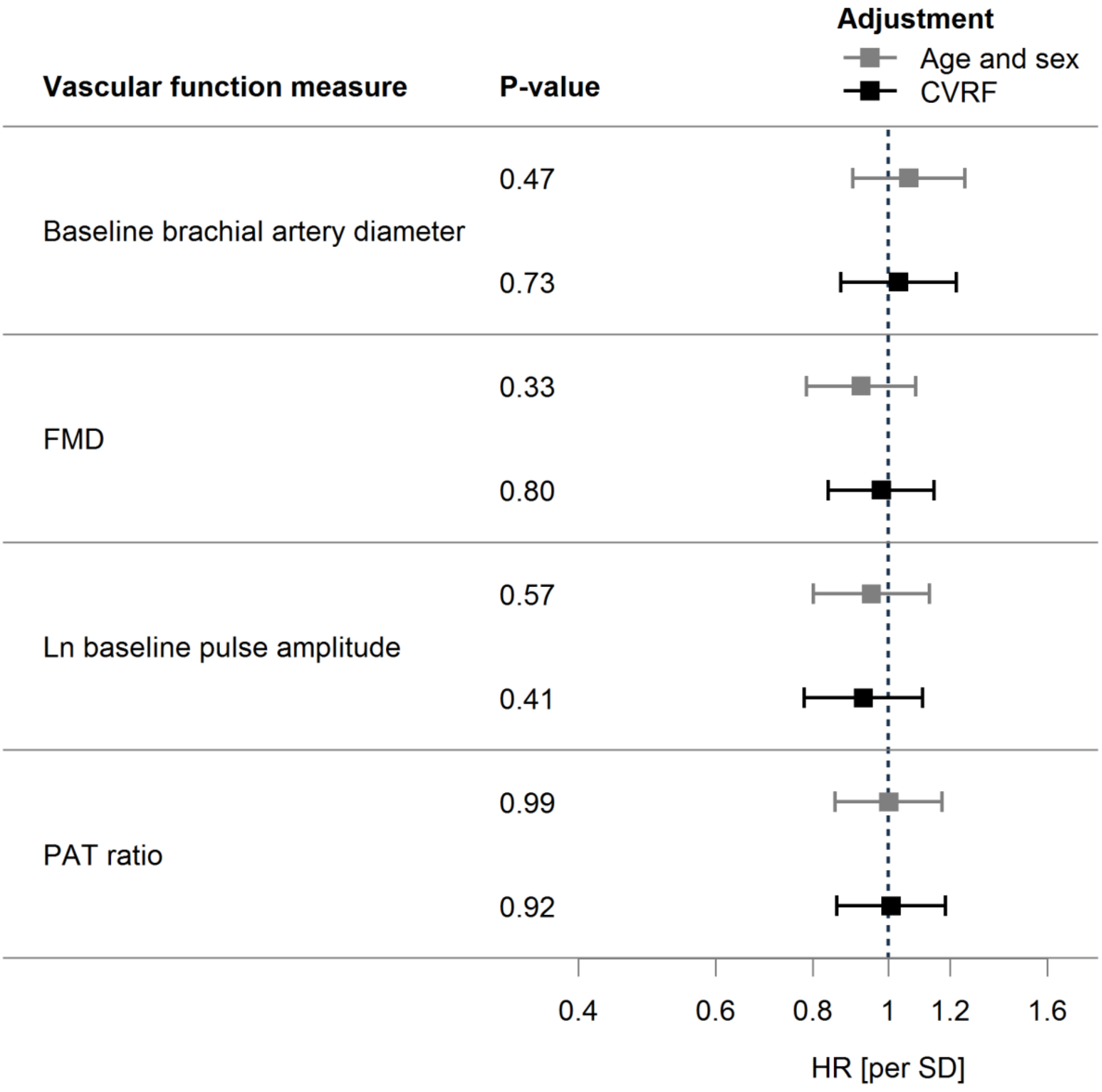

Supplementary Figure 6. Vascular function measures in relation to mortality in multivariable-adjusted Cox regression analyses in the subgroup of individuals with an intermediate ten-year risk of fatal cardiovascular disease (518 events). Provided are hazard ratios (HR) per standard deviation (SD) increase in vascular function measure and 95% confidence intervals.

Age- and sex-adjusted and cardiovascular risk factors (CVRF)-adjusted models are presented. The latter include age, sex, current smoking, body mass index, systolic blood pressure, heart rate, hypertension treatment, diabetes, LDL/HDL cholesterol, lipid treatment, and prevalent cardiovascular disease. FMD stands for flow-mediated dilatation, PAT for peripheral arterial tonometry.

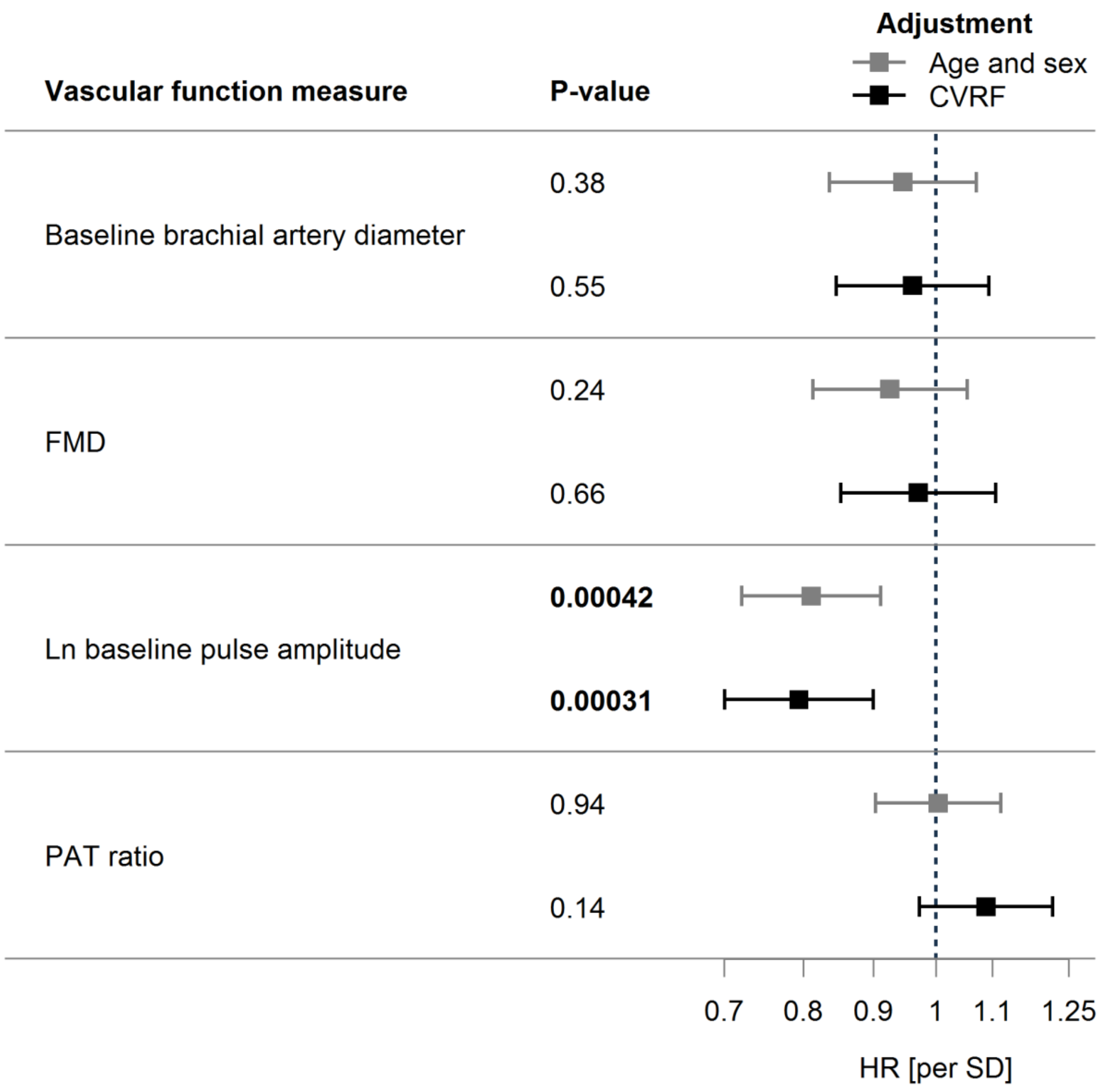

Supplementary Figure 7. Vascular function measures in relation to cardiac mortality in multivariable-adjusted Cox regression analyses in the subgroup of individuals with an intermediate ten-year risk of fatal cardiovascular disease (45 deaths). Provided are hazard ratios (HR) per standard deviation (SD) increase in vascular function measure and 95% confidence intervals.

Age- and sex-adjusted and cardiovascular risk factors (CVRF)-adjusted models are presented. The latter include age, sex, current smoking, body mass index, systolic blood pressure, heart rate, hypertension treatment, diabetes, LDL/HDL cholesterol, lipid treatment, and prevalent cardiovascular disease. FMD stands for flow-mediated dilatation, PAT for peripheral arterial tonometry.

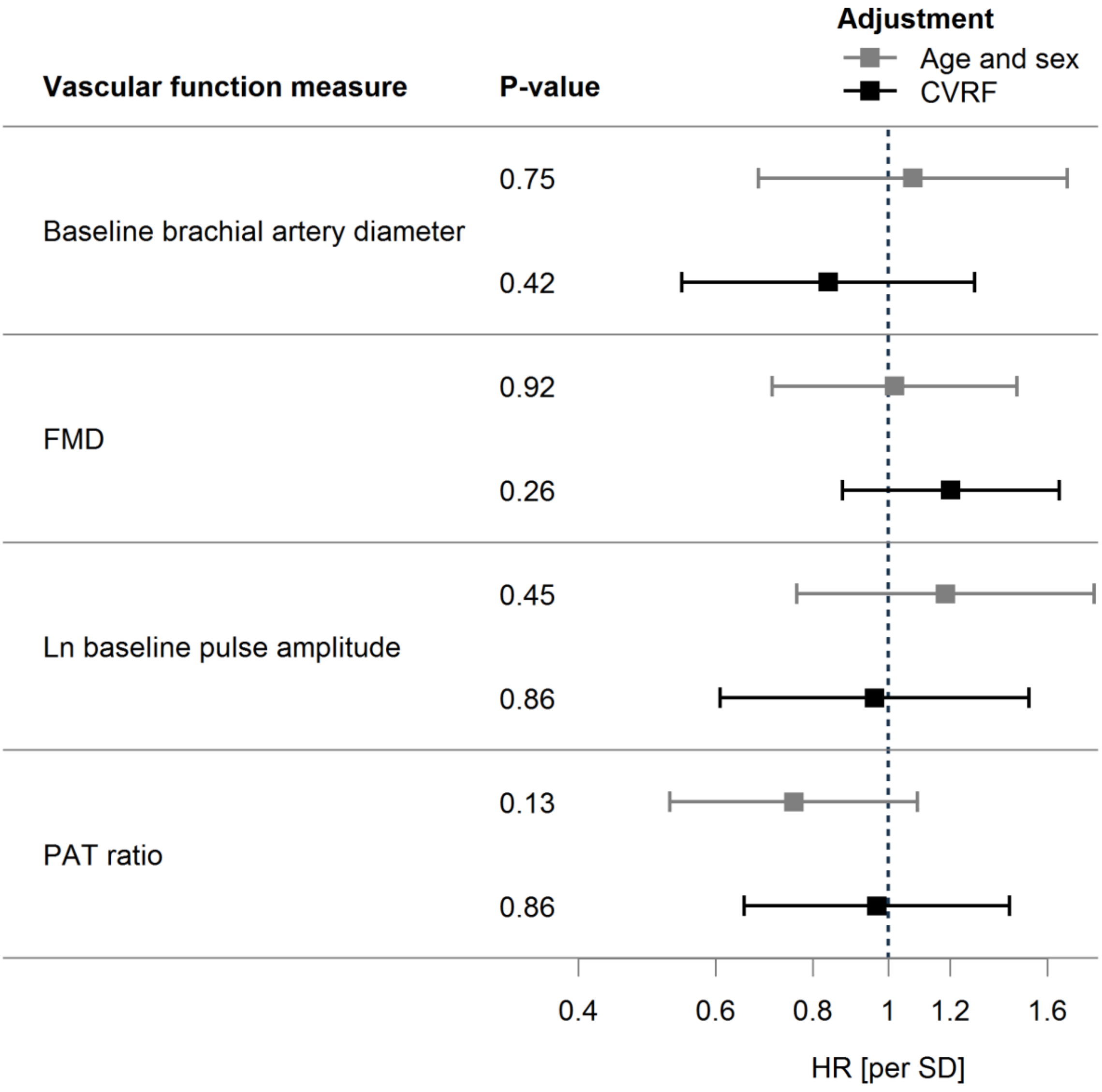

Supplementary Figure 8. Hazard ratios of baseline pulse amplitude plotted against age.

Dashed lines represent 95% confidence intervals. The gray line indicates a hazard ratio of 1.

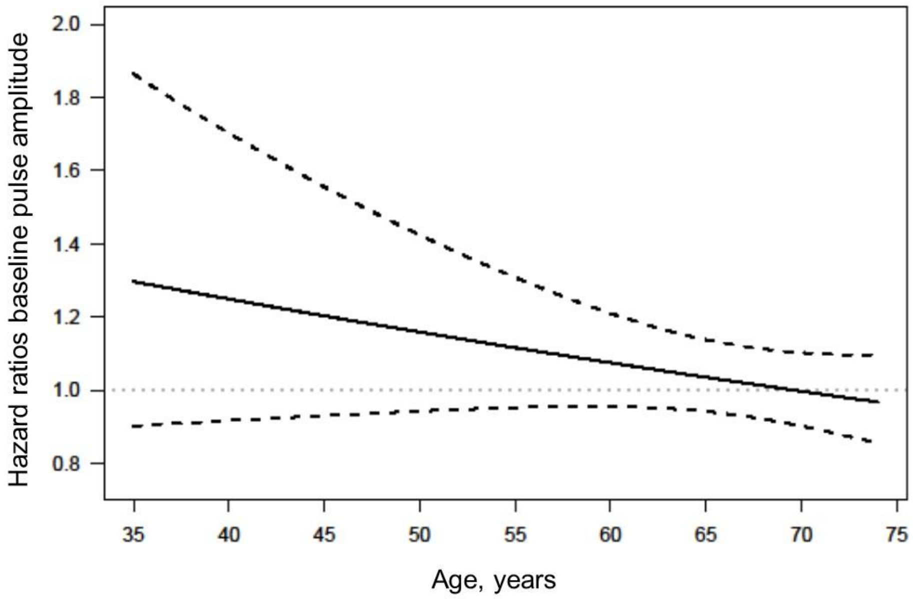

Supplement: cvab087_Supplementary_Data [file cvab087_supplementary_data.pdf]
